# Survey of community pharmacists’ opinions on drug scheduling in Ontario and Québec

**DOI:** 10.1080/20523211.2024.2385936

**Published:** 2024-08-12

**Authors:** Nardine Nakhla, Sherilyn K. D. Houle, Francis Richard, Jeff Taylor

**Affiliations:** a School of Pharmacy, University of Waterloo, Waterloo, Canada; b Faculté de pharmacie, Université de Montréal, Montreal, Canada; c College of Pharmacy and Nutrition, University of Saskatchewan, Saskatoon, Canada

**Keywords:** (MeSH), Community pharmacy services, drug scheduling, self-care, pharmacists, professional role

## Abstract

**Background:**

Over the past decade, Canada has witnessed a shift of several drugs from prescription-only to behind-the-counter (BTC) and over-the-counter (OTC) status. This work examined community pharmacists’ agreement with the current scheduling of agents used in the management of allergic rhinitis, heartburn, and vulvovaginitis.

**Methods:**

From September to October 2022, an online survey was administered to pharmacists practicing in in Ontario and Québec. The survey aimed to gather insights into their preferred scheduling for 15 medicines commonly used to manage the three selected conditions. Pharmacists were asked whether they agreed with the current scheduling status of each and, if not, how they feel it should be scheduled.

**Results:**

715 pharmacists completed the survey, 462 from Ontario and 253 from Québec. Most were staff pharmacists working 30 or more hours per week, having been a pharmacist for 1–10 years. Ontario pharmacists expressed a preference for scheduling change for five drugs (four prescription [terconazole for intravaginal use, famotidine, rupatadine, mometasone nasal spray] and one unscheduled [ranitidine 75 mg]), while Québec pharmacists expressed preference for scheduling change for three prescription drugs (terconazole for intravaginal use, famotidine, rupatadine).

**Discussion:**

As pharmacy practice continues to evolve, pharmacists’ comfort with initiating previously prescription-only therapy independently or assisting patients with self-selection may similarly evolve. Of the five drugs identified as having a discrepancy between current status and pharmacist preference, four are prescription and may be candidates to consider for prescription to OTC switch.

**Conclusion:**

Pharmacists in Ontario and Québec have expressed preference for most products used in the treatment of allergic rhinitis, heartburn, and vulvovaginitis to be categorised as Schedule II (BTC) or Schedule III (OTC) available only in pharmacies.

## Introduction

In Canada, the National Association of Pharmacy Regulatory Authorities [NAPRA], 2022a coordinates a national drug scheduling model, known as the National Drug Schedules (NDS). The NDS aims to standardise the conditions of sale for drugs across the country and consists of four categories – Schedule I (S-I), Schedule II (S-II), Schedule III (S-III), and Unscheduled (U) – that reflect the level of health professional oversight recommended for their safe and effective use (National Association of Pharmacy Regulatory Authorities). S-I drugs require a prescription for dispensing by pharmacists. S-II drugs do not require a prescription but need professional intervention at the point of sale, usually from a pharmacist, and are unavailable for self-selection by the public. S-III drugs are available without a prescription but must be sold in a self-selection area under pharmacist supervision. U drugs can be sold without professional supervision and are available in any retail outlet. Over the years, pharmacists have often taken on more responsibility when agents move off prescriptive status, as have consumers when agents are made more available to them for self-selection on pharmacy shelves and in convenience stores (Consumer Healthcare Products Association, 2023). Pharmacists have noted safety to be the key issue when dealing with such medicines and that their role was to assist patients in making informed choices.(Hanna & Hughes, 2012)

Decisions on where to assign an agent also have important financial ramifications. In 2017, the Conference Board of Canada (Gagnon-Arpin, 2017) estimated that the overall annual economic value of switching three categories of prescribed medicines to non-prescription status would be $1.3 billion (proton pump inhibitors), $767.4 million (oral contraceptives), and $163.0 million (drugs for erectile dysfunction). These figures were based on factors such as avoiding primary care visits and productivity gains (Gagnon-Arpin, 2017).

Understandably, the pharmaceutical industry has a vested interest in this process. Recently, its umbrella organisation implemented a measure called the Self-Care Readiness Index (SCRI, n.d.). The SCRI is claimed to ‘serve as a practical tool for self-care advocates and a catalyst for further debate on the importance of self-care as an effective means to improve the long-term sustainability of health care systems and individual health outcomes, reduce out-of-pocket expenses, and enhance productivity.’ A score on this index will range from 1 (not self-care ready) to 4 (exceptionally self-care ready). In determining a value, they assess stakeholder support, consumer empowerment, self-care health policy, and the regulatory environment of a given country. In its most recent iteration, Canada scored 2.77, Australia was given 3.14, and Mexico garnered a 2.85, whereas in an earlier version of the report, USA and the UK came in at 2.74 and 2.97, respectively. A general tone of the index is that the availability of medicines without prescription is an important component of self-care and it is a path that should be fostered.

For the practice of pharmacy, an area of trepidation continues to center around identifying the agents that should be taken off prescription status, and if (or when) enacted, what level of pharmacy control is most appropriate. In Québec, that is coordinated by the Office des professions du Québec. For the rest of Canada, the task is undertaken by the National Drug Scheduling Advisory Committee (NDSAC). Given ongoing controversies involving some decisions and the existing schedules, NAPRA has evaluated the process several times, including a summit in 2004.

Switching prescribed medicines to over-the-counter (OTC) status is considered an option to improve patient accessibility to treatment, while hopefully ensuring safe and effective use. Accordingly, self-care (and by association, self-medication) is promoted by health officials in many countries (SCRI, n.d.). Pharmacist agreement with scheduling decisions, however, is not a guarantee. In Australia (Mey et al., 2019), pharmacist support for potential reclassifications involved balancing risks and benefits and the potential for misuse when in consumer hands, among other factors. A key driver in making agents more available was a desire to support patient management of minor ailments. Conversely, other pharmacists had concerns over loss of control and revenue, and aspects of patient safety. In Scotland (Paudyal et al., 2014), pharmacists were contacted about four newly reclassified agents – omeprazole, naproxen, simvastatin, and chloramphenicol eye drops. Adopting the agents into actual patient management showed varying degrees of uptake. Support was high in general, but less for OTC simvastatin due to a perceived lack of efficacy at OTC doses and low patient demand.

A decade ago, a survey of Canadian pharmacists (Taylor et al., 2012) determined they generally favored tighter control of OTC agents, especially those classified as U (e.g. small bottles of dextromethorphan syrup, miconazole foot cream, ranitidine 75 mg), although some provincial differences were in play. There was a sense that some decisions may have been too aggressive, and this could reflect a potential disconnect between regulators – those deciding how OTCs should be legislatively categorised in Canada – and front-line pharmacists. In that series of reports, pharmacists (at the time) did not support the deregulation of simvastatin nor omeprazole and only moderately supported an OTC switch for intranasal fluticasone. The complexity of disease and drug management was listed as the main barriers by those who were against such a switch, while additional training was deemed necessary by pharmacists who were supportive (Lalonde et al., 2012).

The objective of this report is to outline the level of community pharmacist support for current drug scheduling in Ontario and Québec, given notable differences in drug scheduling status between them, for agents used in the management of allergic rhinitis, heartburn, and vulvovaginitis.

The rationale for this research stems from the critical role that community pharmacists play in medication management and patient care. As frontline healthcare professionals, pharmacists are entrusted with the responsibility of ensuring the safe and effective use of medications. However, discrepancies between drug scheduling regulations and pharmacist perspectives can pose challenges to this responsibility. Understanding pharmacists’ opinions on drug scheduling is therefore essential for aligning regulatory policies with practice realities and optimising patient outcomes. Despite the importance of this issue, existing research often lacks a comprehensive understanding of pharmacist perspectives and the factors influencing their opinions on drug scheduling. By addressing this knowledge gap, our study aims to provide valuable insights that can inform policy decisions, enhance pharmacist training and practice, and ultimately improve the quality of pharmaceutical care delivery.

Through this exploration, our research may contribute to better understanding of drug scheduling challenges not only in Canada but internationally, where similar issues in balancing regulatory policies with pharmacist perspectives and patient needs arise. Additionally, our comparative analysis of regional variations within a single country enables international readers to draw parallels with their own regulatory frameworks and pharmacist perspectives, fostering cross-cultural understanding and knowledge exchange.

## Methods

### Population of interest

This was a cross-sectional survey of practicing community pharmacists in Ontario (n = 11,658) and Québec (n = 7,258) (National Association of Pharmacy Regulatory Authorities, 2022b). Target quotas for each province were set at 308 (Ontario) and 192 (Québec) to align with the relative proportion of licensed community pharmacists in each province. Pharmacists practicing less than 1 h per week in community pharmacy in Ontario or Québec were excluded from the study.

By selecting Ontario and Quebec for our study, we aimed to capture the diversity in drug scheduling practices across the two largest provinces in Canada. These provinces were chosen specifically because they differ the most in terms of their regulatory frameworks and decision-making processes related to drug scheduling. This allowed us to explore variations in pharmacist opinions and preferences within the context of distinct provincial regulations.

### Questionnaire design

Many reports have examined medicine re-classification in various parts of the world and these provided guidance for the process (Chang et al., 2016; Gauld et al., 2015; Mey et al., 2019; Nomura et al., 2016; Paudyal et al., 2012; Sega & Sullivan, 2011; Stippler et al., 2019). Approaches taken have included the tendency to make agents available to the public (Gilbert et al., 2006), gauging support for a behind-the-counter category (Sega & Sullivan, 2011), and presenting lists of potential switch candidates to see whether pharmacists support the move (Mey et al., 2019; Paudyal et al., 2014; Stippler et al., 2019). However, a pre-existing questionnaire to meet all study objectives was not found in the literature. One Canadian report (Taylor et al., 2012) was similar in intent, and it formed the basis of the document. It included 25 OTC medicines across a variety of scheduling requirements, where pharmacists were asked whether they agreed with the current scheduling status and if not, how the agent should be scheduled (S-I, S-II, S-III, or U). For the current report, 15 agents were selected that are used in the management of heartburn, allergic rhinitis, and vulvovaginitis, where notable provincial differences exist. Demographic survey items such as gender and practice setting were similar to the previous report.

The final questionnaire was pre-tested among 7 pharmacists and pharmacy practice researchers who completed the questionnaire and provided feedback on its clarity, and minor modifications were made. Scale validity was not deemed to be relevant, given that the measure was a simple vote. Reliability testing (e.g. voting for S-I initially, but then changing to S-III a few weeks later) was not carried out.

The survey was available in both English and French and was administered online by the Survey Research Centre at the University of Waterloo.

### Questionnaire delivery

Email addresses of practicing community pharmacists in Ontario (who indicated a willingness to be contacted for research purposes) was obtained from the Ontario College of Pharmacists. All pharmacists in this database were sent an advance email (Sept 12, 2022), which explained the nature of the survey and what was being asked and why. This was followed by the main email contact (Sept 20, 2022) that again outlined the intent and provided a unique closed URL to the survey. Two email reminders were sent to non-respondents to help maximise survey response rates (Oct 3 and 12, 2022). In Québec, an email address list was not available, so social media platforms (Facebook and LinkedIn) were used to advertise the survey, with an open (non-unique) URL link provided.

To minimise the risk of automated/fraudulent completion, CAPTCHA (Completely Automated Public Turing test to tell Computers and Humans Apart) was utilised, as well as mandatory randomised questions designed to catch bots. Metadata was also captured and reviewed to identify duplicate IP addresses. Completed questionnaires were adjudicated for authenticity, completeness, and target audience by the Survey Research Centre.

An incentive of a $5 Starbucks e-gift card was provided for each valid completed survey to those respondents who chose to accept one.

### Analysis

The Statistical Package for the Social Sciences (v28) was used for descriptive analysis. In addition, a Dissatisfaction Index was calculated as a measure of total votes *against* the status quo divided by the total votes *for* the status quo. A value of 1 would be neutral – equal numbers of votes on either side. Numbers rising above 1.0 would be a marker for a degree of dissatisfaction. By way of example, a value of 3.0 would result if 300 votes were received against the incumbent (total of the other three categories) compared to 100 votes for the incumbent schedule.

### Ethics approval

All identifying information (email and IP addresses) were removed from the final data file to ensure confidentiality of the respondents. The University of Waterloo Research Ethics Board approved the study on ethical grounds in Aug 2022 (# 44519).

## Results

Data collection took place from September 20 to October 19, 2022. A total of 6,507 initial survey invitations and 12,205 email reminders were sent to Ontario pharmacists. At survey close, 715 pharmacists completed the survey, with 462 from Ontario and 253 from Québec. A definitive response rate (as defined by the American Association for Public Opinion Research) could not be calculated, as it was not possible to accurately identify the total number of people who were made aware of the survey on social media. Sampling weights were not applied to the data, since the proportion of respondents from each province across the sample and population were almost identical. This holds true for both the number of community pharmacists and all licensed pharmacists in each province. As such, unweighted data was used for analysis. The median time for survey completion was 12 min.

### Respondent characteristics

The demographics of respondents and information on their primary practice site are summarised in Table 1. There were proportionally more female respondents from Québec (58.2%) than Ontario (46.2%). Most were working 30 or more hours per week and have been a pharmacist for 1–10 years, most commonly as a staff pharmacist. At the pharmacy designated as their main location of employment, most were in large urban areas. Independent pharmacies were more common in Ontario, with banner/franchise outlets being of note in both provinces. *Steady (moderate pace)* and *busy with slow periods* reflected the most common descriptions for what a typical day was like for responding pharmacists.

**Table 1.** Respondent characteristics.

	Ontario (n = 462)	Québec (n = 253)
**Number of years in practice**		
Less than 1 year	7	19
1–5 years	135	84
6–10 years	142	70
11–15 years	69	39
16–20 years	25	18
More than 20 years	84	23
**Hours per week worked in a community pharmacy, on average**		
1-14 h/week	70	39
15-29 h/week	104	50
30-39 h/week	191	134
40 h or more	97	30
**Role at the community pharmacy**		
Pharmacy owner	77	21
Pharmacy manager/Chief pharmacist	130	37
Staff pharmacist	232	178
Locum/relief pharmacist	73	47
Other	3	0
**Community population**		
Large urban (100,000 or more)	221	112
Medium center (30,000–99,999)	162	86
Small center (1,000–29,999)	74	49
Rural (less than 1,000)	5	6
**Pharmacy type**		
Independent	174	20
Chain associated with a supermarket (e.g. Loblaws, Sobeys)	67	25
Mass merchant/discount retailer (e.g. Costco, Walmart)	86	36
Banner/Franchise store (e.g. Shoppers Drug Mart, Pharmasave, PharmaChoice, Jean Coutu, Uniprix)	124	170
Other	11	2
**Pharmacy pace in a typical day**		
Slow	21	10
Slow with busy periods	90	39
Steady moderate pace	153	74
Busy with slow periods	103	84
Busy	95	46

### Drug scheduling

Tables 2 and 3 list the current drug scheduling status for various entities in the two provinces, and the corresponding level of support for that category. Ontario pharmacists were more likely to disagree with existing categories than their Québec counterparts. Relative to the Dissatisfaction Index, Ontario pharmacists voted for status change for five different drugs, with a sixth (omeprazole) at the break-even point. Québec pharmacists proposed changing the status of three, with the Dissatisfaction Index being notable for famotidine and rupatadine. Ranitidine garnered the most support for Unscheduled status in Ontario (23.2%), whereas there was minimal appetite for that outcome for any agent in Québec.

**Table 2.** Drug scheduling and pharmacist agreement in Ontario (n = 462).

Drug product	Current status*	What status should it be?				
		I	II	III	U	Dissatisfaction index
**Drugs for the management of vulvovaginal candidiasis**						
Terconazole for intravaginal use	I	184	160	114	4	1.5
Clotrimazole for intravaginal use	III	30	108	298	26	0.6
Miconazole for intravaginal use	III	37	87	316	22	0.5
Fluconazole 150 mg when sold as 1 capsule package size	III	32	130	279	21	0.7
Fluconazole 150 mg capsule + clotrimazole external cream	III	24	124	294	19	0.6
**Drugs for the management of heartburn**						
Esomeprazole 20 mg when sold as 14-day treatment package	III	29	162	258	13	0.8
Omeprazole 20 mg when sold as 14-day treatment package	II	35	229	182	16	1.0
Famotidine 40 mg	I	115	183	140	24	3.0
Ranitidine 75 mg when sold in package sizes containing ≤4500 mg	U	44	126	185	107	3.3
**Drugs for the management of allergic rhinitis**						
Rupatadine 10 mg	I	153	194	99	16	2.0
Diphenhydramine 25 mg	III	26	111	293	32	0.6
Loratadine 5 mg + Pseudoephedrine 120 mg	III	24	137	277	24	0.7
Fluticasone propionate 50 mcg intranasal spray when sold as package sizes containing no more than 360 metered sprays for use in ages 18 and up	III	29	129	285	19	0.6
Mometasone 50 mcg intranasal spray for use in ages 12 and up	I	114	176	155	17	3.1
Triamcinolone 55 mcg intranasal spray when sold as package sizes containing no more than 120 metered sprays for use in ages 12 and up	III	31	148	273	10	0.7

*At the time the survey was created.

**Table 3.** Drug scheduling and pharmacist agreement in Québec (n = 253).

Drug product	Current status*	What status should it be?				
		I	II	III	U	Dissatisfaction index
**Drugs for the management of vulvovaginal candidiasis**						
Terconazole for intravaginal use	I	102	112	38	1	1.5
Clotrimazole for intravaginal use	III	14	51	185	3	0.4
Miconazole for intravaginal use	III	18	37	195	3	0.3
Fluconazole 150 mg when sold as 1 capsule package size	II	13	183	51	6	0.4
Fluconazole 150 mg capsule + clotrimazole external cream	II	15	184	47	7	0.4
**Drugs for the management of heartburn**						
Esomeprazole 20 mg when sold as 14-day treatment package	II	15	168	66	4	0.5
Omeprazole 20 mg when sold as 14-day treatment package	II	14	168	64	7	0.5
Famotidine 40 mg	I	46	122	79	6	4.5
Ranitidine 75 mg	III	8	54	187	4	0.4
**Drugs for the management of allergic rhinitis**						
Rupatadine 10 mg	I	60	134	54	5	3.2
Diphenhydramine 25 mg	III	20	55	171	7	0.5
Loratadine 5 mg + Pseudoephedrine 120 mg when sold as package sizes containing more than 10 tablets	II	8	158	82	5	0.6
Fluticasone propionate 50 mcg intranasal spray for use in ages 18 and up	II	12	161	76	4	0.6
Mometasone 50 mcg intranasal spray for use in ages 12 and up	II	16	173	58	6	0.5
Triamcinolone 55 mcg intranasal spray for use in ages 12 and up	II	14	161	75	3	0.6

*At the time the survey was created.

## Discussion

This report surveyed pharmacists to garner feedback on the existing drug scheduling legislation applicable to 15 agents across three therapeutic areas. Where there was less support for the current dynamics, it allowed pharmacists to state where such agents would be ‘better’ positioned for sale. Where there was more support, it provides a degree of confirmation that legislators essentially got it right (in the opinion of those pharmacists), such as over two-thirds supporting S-III status for intravaginal miconazole in both provinces.

Overall, Ontario pharmacists were more likely to disagree with a variety of existing classifications compared to Québec pharmacists, which may be reflective of inherently greater control during sale in that province. Seven agents are classified in higher categories in Québec, while one (mometasone) in Ontario went against that trend.

Québec pharmacists appeared to be more decisive in their voting, across almost all agents (except terconazole where support for Schedule I and II was similar). Conversely, Ontario pharmacists showed relatively similar support across three schedules for terconazole, famotidine, rupatadine, and mometasone. There was slightly more support for U sales in Ontario, with a notable one being ranitidine (23.2 percent in favour). In Québec, only 1.6 percent would support such a move for that agent.

What this tells us is that pharmacists clearly differ on the approach to take, for decisions involving three therapeutic categories. Thus, the ability of an authoritative body (like NDSAC) to reach consensus during decision-making is a lot to expect. But many challenging decisions are made every day in society, such as mask-wearing laws during COVID or speed limits around parks and schools; support will be divided. Whether that should instigate providing more (or better) background to end users can be debated. It may also bring into question the communication methods currently in place for the delivery of it. Either way, some discord is evident and the extent that it might affect practice behaviour would be of great interest.

The results may have identified professional cultural differences in the provinces. A lack of support for U status in Québec may suggest less confidence in the public’s ability to use agents appropriately without pharmacy intervention. A remedy for that would mean (at a minimum) a move to S-III, and although that places a pharmacist in the vicinity of a sale, it does not guarantee consumers will ask for help. The vast majority of OTC consults involving S-III agents occur when the patient ASKS for help, not pharmacists offering it. Therefore, Québecers may have more faith in the existence of the S-III category.

Reasons as to why pharmacists voted as they did were not obtained, however. Further speculation on the possibilities include: some pharmacists might see less need for MD intervention (an S-I agent should be moved down to S-II), a concern over physician access, some agents do not require the direct involvement of a pharmacist (S-IIs moved down to S-III), a move to improve patient safety (S-IIIs up to S-II), concerns that the public cannot safely self-medicate (Us up to S-III), and for insurance plan coverage (S-II/S-IIIs up to S-I).

The first time this was assessed in Canada was over a decade ago (Taylor et al., 2012). There was strong support for ASA 81 mg tabs, minoxidil 2% topical solution, hydrocortisone 0.5% cream, and BP 5% lotion to be retained at Schedule III. Ferrous sulphate 300 mg tablets should be kept as Schedule II. Overall, it showed that pharmacists generally favoured tighter control of OTC agents, particularly those that were unscheduled at the time (although provincial differences existed). About a quarter supported the current U status, while two-thirds would support a jump to S-III. For acetaminophen 325 mg tabs, 55.4 percent supported the current U, while 43.0 percent would opt for S-III. Reasons for the voting was not obtained then either. Ten years hence, pharmacists still do not wholly support the notion of U status for OTC medicines, albeit for more specific areas this time.

In this new report, there was notable support for S-II classifications in Québec, ones that obviously call for more pharmacist intervention. In the United States for perspective, a recent FDA ruling would not create a BTC category of drugs (Rumore, 2022). In Australia, the equivalent schedule requires a pharmacist’s involvement in their sale to ascertain the therapeutic need for the medication (Emmerton, 2009). During observations involving those agents in pharmacies, interaction took place in only 54.7 percent of sales. A system has been in place to monitor quality standards since 2002 (Benrimoj et al., 2008). A systematic review (van Eikenhorst et al., 2017) of pharmacist consultations (mostly in the UK) involving OTC medicines found that advice was not consistently offered.

The limitations of this research, consistent with survey-based methodology, acknowledge the challenge of condensing complex human behavior into numerical constructs. While surveys efficiently capture attitudes and perceptions, they risk oversimplification, potentially overlooking nuances best captured through qualitative methods. Response bias, recall inaccuracies, and unexplored factors such as individual characteristics and regional variations in healthcare delivery further underscore the need for cautious interpretation of our findings. Additionally, as a voluntary survey, the demographics of non-respondents and reasons for non-participation among those opting not to complete the survey could not be collected. Lastly, while targets were met for sample size, there are no guarantees they represent the population of pharmacists, particularly those in jurisdictions other than the ones studied.

Our study provides a careful examination of distinct approaches to managing minor ailments among pharmacists in Ontario and Québec, revealing variations both within and across these populous Canadian provinces. These findings offer valuable insights into the diversity of therapeutic choices to manage each condition, stimulating discourse regarding optimal care strategies. Furthermore, our findings underscore pharmacists’ notable level of confidence in addressing these cases, reflecting their expertise and preparedness in managing four common ailments. Additionally, our study offers valuable perspectives on how regulatory changes may influence pharmacists’ therapeutic approaches, highlighting the significance of drug scheduling on patient care decision-making and prompting consideration of optimal therapeutic and regulatory approaches.

This research has significant implications for improving practice and policy in Canada. By elucidating the variations in pharmacists’ approaches and confidence levels, our findings can inform the development of targeted training programs and guidelines to enhance consistency and quality of care across provinces. For readers outside of Canada, this study provides a model for understanding how regional regulatory differences can impact healthcare delivery, offering lessons that can be applied to other contexts. The insights gained here are particularly useful for policymakers, educators, and healthcare professionals looking to optimise therapeutic strategies and regulatory frameworks in diverse settings.

While our study provides insights into pharmacist opinions on drug scheduling in Ontario and Québec, caution should be exercised in generalising these findings nationally and internationally due to potential variability in practice and healthcare delivery across provinces and countries. However, our research, focused on community pharmacists in the two largest provinces in the country, may unveil insights with broader implications for international readers. Drug scheduling and pharmacist opinions pose challenges not unique to Canada, offering valuable lessons for healthcare systems worldwide. By comparing regional perspectives within Canada, our study facilitates cross-cultural understanding and knowledge exchange, providing international readers with parallels to their own regulatory frameworks. Furthermore, our findings highlight some factors influencing pharmacist opinions and their impact on patient care, informing global discussions on pharmaceutical care delivery and underscoring the international relevance of our study's outcomes.

## Conclusion

How agents are scheduled has important implications, both clinically and economically. This survey offers some uniqueness to pharmacy practice in that it allowed community pharmacists to either support existing parameters, or state where such agents would be better positioned for sale. Feedback on the level of support for existing drug scheduling legislation across 15 agents revealed notable differences not only between provinces, but also among pharmacists within Ontario. This diversity of opinions underscores the ongoing challenge of achieving any degree of consensus for a regulatory body. To further our understanding and address this complexity, we recommend future research initiatives tackle the nuances and variations in pharmacist perspectives on drug scheduling. Qualitative studies can delve into the underlying factors influencing pharmacist opinions, particularly in regions with diverse regulatory frameworks. Longitudinal studies are essential for tracking evolving trends in pharmacist perspectives over time. Additionally, assessing the impact of drug scheduling changes on patient outcomes is crucial for evidence-based decision-making and ensuring patient safety. Moreover, fostering stakeholder collaboration will facilitate inclusive decision-making processes aligned with patient needs, thereby optimising medication access and safety nationwide.

## Roles

NN and JT conceived, initiated, and supervised the project; JT wrote the initial draft of manuscript; NN, JT, SH, and FR contributed to the questionnaire development and collected the data; all authors contributed to result synthesis and writing of the manuscript; NN finalised the manuscript with all author approvals.
